# Hexavalent Chromium Removal from Model Water and Car Shock Absorber Factory Effluent by Nanofiltration and Reverse Osmosis Membrane

**DOI:** 10.1155/2017/7415708

**Published:** 2017-07-27

**Authors:** Amine Mnif, Imen Bejaoui, Meral Mouelhi, Béchir Hamrouni

**Affiliations:** Desalination and Water Treatment Research Unit, Faculty of Sciences of Tunis, University of Tunis El Manar, El Manar II, 2092 Tunis, Tunisia

## Abstract

Nanofiltration and reverse osmosis are investigated as a possible alternative to the conventional methods of Cr(VI) removal from model water and industrial effluent. The influences of feed concentration, water recovery, pH, and the coexisting anions were studied. The results have shown that retention rates of hexavalent chromium can reach 99.7% using nanofiltration membrane (NF-HL) and vary from 85 to 99.9% using reverse osmosis membrane (RO-SG) depending upon the composition of the solution and operating conditions. This work was also extended to investigate the separation of Cr(VI) from car shock absorber factory effluent. The use of these membranes is very promising for Cr(VI) water treatment and desalting industry effluent. Spiegler-Kedem model was applied to experimental results in the aim to determine phenomenological parameters, the reflection coefficient of the membrane (*σ*), and the solute permeability coefficient (*P*_*s*_). The convective and diffusive parts of the mass transfer were quantified with predominance of the diffusive contribution.

## 1. Introduction

The rapid industrialization and development in industrial processes led to the presence of heavy metals in water and industrial effluents. These components are extremely toxic and are released in huge quantities by several industries. Among these constituents, one can mention the chromium compounds that exist in several oxidation states as trivalent chromium (Cr(III)) and hexavalent chromium (Cr(VI)).

Cr(III) is considered as an essential trace nutrient for humans with an established adequate daily dietary intake range for adults from 20 to 35 *μ*g chromium [1]. However, high concentration of this element is extremely toxic, teratogenic, and mutagenic presenting a great threat to human beings when ingested through the respiratory and digestive tract or through skin contact [2, 3]. In addition, Cr(VI) is included in the list of the US Environmental Protection Agency for prioritizing control of its application. According to the World Health Organization standards, the maximum level for chromium is 0.05 mg L^−1^ for hexavalent chromium and 0.1 mg L^−1^ for total chromium in drinking water [4, 5].

Chromium is extensively used in tanning operation to obtain leather of desirable quality. It is also used in the production of pigments, the manufacture of stainless steel, and electroplating and as a biocide in the cooling waters of nuclear power plants [6].

Among the nine valence states of chromium ranging from −2 to +6, only hexavalent and trivalent chromium have primary environmental significance due to their stable oxidation forms in the environment [7]. Depending on the pH of the solution, Cr(VI) typically exists in two forms, chromate (CrO_4_^2−^) and dichromate (Cr_2_O_7_^2−^) [8]. These two divalent oxyanions are very water soluble and poorly adsorbed by soil and organic matter, making them mobile in soil and groundwater [9].

Therefore, in order to sustain our global water supply, analytical methods for the removal of Cr(VI) were developed such as chemical reduction, precipitation, evaporation, and ion exchange [10–12]. Although ion exchange resins can substantially remove metal ions, they do not show mechanical strength because of swelling of polymeric skeleton and low selectivity. Precipitation is high cost process and treated water still has high chromium ion concentrations. These traditional methods have certain drawbacks and considerable attention has been focused upon absorption and membrane separation [13, 14].

Membrane separation has become increasingly attractive for treatment and recovery of heavy metals due to its high efficiency, ease of operating, and low cost [15, 16].

In this study, we report the behavior and efficiency of reverse osmosis and nanofiltration on the removal of Cr(VI) under different water quality conditions, such as pH, ionic strength, and the presence of different competing elements. This work was also extended to investigate the separation of Cr(VI) from car shock absorber factory effluent in order to meet the environmental local limits (<5 mg L^−1^) before discharging into the municipal treatment plant. The model of Spiegler-Kedem was then applied to determine the phenomenological and mass transfer parameters.

## 2. Materials and Methods

The present work was performed on a pilot plant equipped with commercial spiral wound reverse osmosis membrane RO-SG-2514 and nanofiltration membrane NF-HL-2514 supplied by Osmonics. Both modules used for this study are approximately 64 mm in diameter and 356 mm in length.

**Figure 1:**
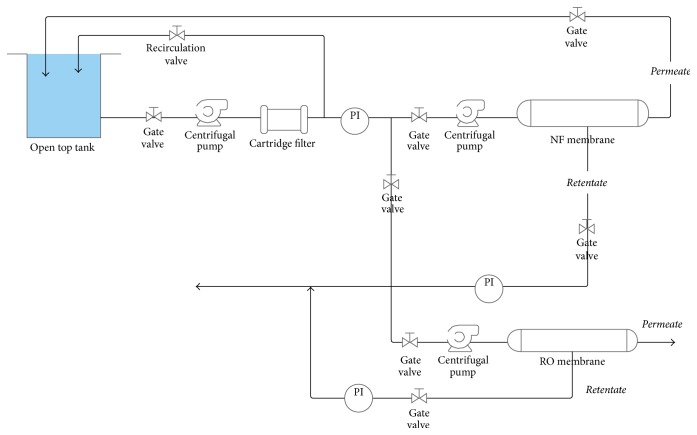
Schematic diagram of test system.

Figure 1 shows the schematic diagram of the experimental set-up used in this study. Permeate and retentate water was recycled to the feed tank in order to keep the concentration of the feed solution stable.

The specifications of the membranes in the pilot scale system are described in Table 1.

Before all experiments, the membranes were cleaned and rinsed with ultrapure water (0.05 *μ*S cm^−1^) at *P* = 5 bar for at least 30 minutes. The permeate flux *J*_*v*_ was determined by measuring the volume of permeate collected in a given time interval per unit membrane area. Observed rejection was calculated by

(1) $$\begin{array}{c} R=\left(\;1-\frac{C_{p}}{C_{0}}\;\right)\cdot 100, \end{array}$$

where *C*_*p*_ and *C*_0_ are permeate and feed concentrations, respectively.

The conversion rate *Y* is given as

(2) $$\begin{array}{c} Y=\left(\;\frac{Q_{p}}{Q_{0}}\;\right)\cdot 100, \end{array}$$

where *Q*_0_ and *Q*_*p*_ are the initial and permeate flow rates, respectively.

The phenomenological parameters (salt permeability (*Ps*) and reflection coefficient (*σ*)) were determined using the model of Kedem–Katchalsky [17].

NaCl (99.5%, Sigma Aldrich), CaCl_2_ (99.8%, PROLABO), and Na_2_SO_4_ (99.0%, ACROS) concentrations and wastewater salinity were measured by conductivity. Concentration of Cr(VI) in the permeate was determined using a UV-Vis spectrophotometer (TOMOS V-1100) at 540 nm wave length with 1,5-diphenylcarbazide as a color complexing agent [18]. The adjustment of pH was made using NaOH and HCl. All the chemicals used in this study were of analytical grade from Merck. The chromium solutions were prepared using distilled water.

To evaluate the charge of the two membranes, salt retention measurements with CaCl_2_, NaCl, and Na_2_SO_4_ (10^−3^ mol L^−1^) as a function of permeate flux give the following retention sequence: *R*_Na_2_SO_4__ > *R*_CaCl_2__ > *R*_NaCl_, which is caused by differences in diffusion coefficients between the different salts for both membranes [19]. However, previous works showed a negatively charged character of both membrane surfaces [20, 21].

## 3. Results and Discussion

### 3.1. Retention of Hexavalent Chromium Ions from Model Water

#### 3.1.1. Effect of Conversion Rates on Hexavalent Chromium Retention

**Figure 2:**
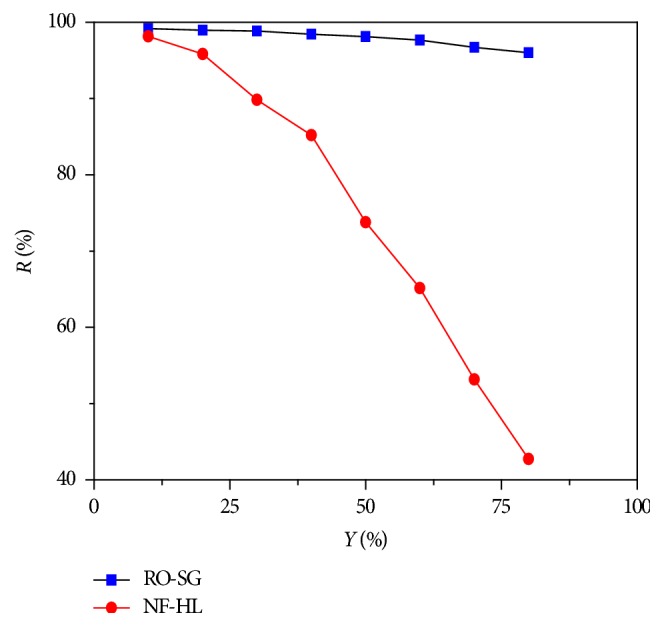
Retention rates of hexavalent chromium as a function of conversion rate (Δ*P* = 6 bar, *C*_Cr(VI)_ = 11 mg L^−1^, pH = 8, and *θ* = 25°C).

The retention rate of Cr(VI) was studied as a function of conversion rate. An increase in the conversion rate caused a decrease in the retention rate under the same pressure (Figure 2); this could be the result of the decrease in cross flow velocity and the appearance of polarization layer with high conversion rates [22–24].

#### 3.1.2. Effect of Initial Concentration and pH on the Retention Rate of Hexavalent Chromium

**Figure 3:**
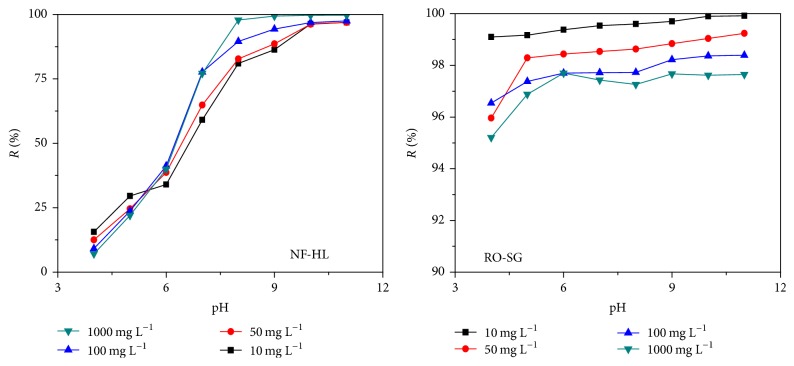
Evolution of retention rate of Cr(VI) as a function of initial feed concentration at different pH.

The experiments were carried out on solutions of K_2_Cr_2_O_7_ with concentration ranging between 10 and 1000 mg L^−1^ at various pH for a fixed transmembrane pressure of 7 bar for NF-HL and RO-SG (Figure 3).

The retention rate of the Cr(VI) changed according to the variation of feed pH as well as concentration, but each membrane shows a particular behavior.

Thus, in the acidic range, more retention rate was observed at lower initial chromium concentration compared to higher concentration for NF-HL membrane (30 to 20% at pH 5), but passing to alkaline range, a reverse trend was observed, where higher retention rate occurred at higher concentrations (80.0 to 99.7% at pH 8) which can be explained by the dielectric exclusion effect [20]. In fact, Cr(VI) can exist in the aqueous solution in different ionic forms (HCrO_4_^−^, CrO_4_^2−^, Cr_2_O_7_^2−^), which depend on Cr(VI) concentration and solution pH [25, 26]. According to these results, one can conclude that, in the pH range 4–5.5, the possible form of Cr(VI) according to the concentrations is HCrO_4_^−^, and in the pH range of 5.5–11, the various possible forms are HCrO_4_^−^ until pH 6.5 and CrO_4_^2−^ until pH 11. Therefore, the decrease of the retention rate of Cr(VI) between pH 4 and 5.5 cannot be explained by a change in chromium form but is presumably due to the screening effect caused by the presence of the negative charge on the NF-HL membrane surface [17]. Passing from pH 5.5 to 11, the increase of retention rate with concentration could be explained by the dielectric exclusion effect besides the change in chromium forms due to the changes in the relative amount of monovalent (HCrO_4_^−^) and divalent ions (CrO_4_^2−^) present in solution. This can explain the higher retention at high concentration in the studied pH range when dissociation to bivalent ions is more extensive.

Whereas the retention rate of Cr(VI) decreased all over the range of pH when increasing concentration for RO-SG membrane (99.9 to 97.0% at pH 8), this can be explained by the screening effect involved by the increase in the concentration of potassium cations in the solution [27]. At high salt concentration (K_2_Cr_2_O_7_), the negative charge of the membrane was gradually neutralized by potassium cations and therefore a decrease in the electrostatic repulsions (between the anions in solution and the negative charges of the membrane) facilitated the transfer of HCrO_4_^−^ or CrO_4_^2−^ ions through the membrane.

An increase in retention rate of Cr(VI) with increasing pH values for each concentration was observed for both membranes. In fact, in a pH below 6.5, monovalent species HCrO_4_^−^ were dominant, but when pH was higher than 6.5, divalent species CrO_4_^2−^ were dominant.

For pH ranging from 4 to 6.5, almost all the chromium species passed through the membrane. When the pH was adjusted to 6.5, the permeate concentration of HCrO_4_^−^ became lower due to the fact that 50% of the ions were converted to CrO_4_^2−^ when pH = pKa. When the pH was adjusted from 8 to 11, nearly complete removal was achieved, resulting from all the chromium species becoming CrO_4_^2−^. This behavior is also in a good agreement with the literature [26, 28, 29].

#### 3.1.3. Effects of Coexisting Ions

Since real solutions contain different types of ions at different concentrations, possible interaction with the surface of membranes may affect the removal efficiency of Cr(VI). The correlations between the Cr(VI) removal and the coexisting ions simulated for different ionic strength in feed water were investigated.

**Figure 4:**
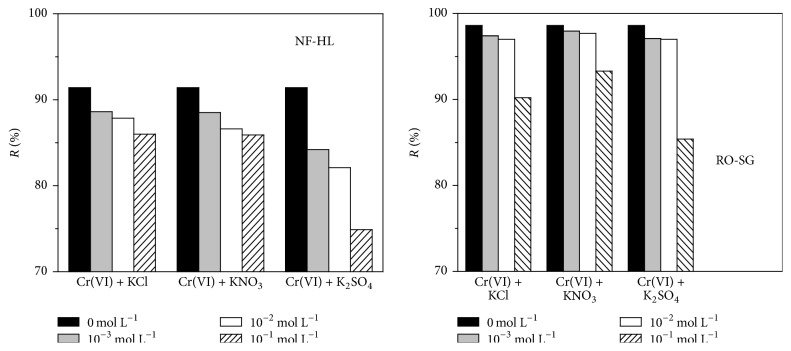
Effect of the ionic strength and coexisting ions on the retention rate of Cr(VI) (*C*_Cr(VI)_ = 50 mg L^−1^, *Y* = 50%, Δ*P* = 6 bars, pH = 8, and *θ* = 25°C).

The effect of different competitive ions such as chloride, nitrate, and sulfate on the retention rate of Cr(VI) was studied at various concentrations for both membranes (Figure 4).

The results show that the retention rate of Cr(VI) decreased with increasing salt concentrations since the electrostatic effects of the membrane become weaker as KCl, KNO_3_, or K_2_SO_4_ concentration increases. This is known as the screening phenomenon, where potassium ions neutralize partially the negative charges of the membrane, which involves the decrease of the retention of charged ions, thus facilitating the passage of the hexavalent chromium ions [26]. This was later reported by Hafiane et al. [28]. They mentioned that this can be related to the dependence of the effective charge density (Φ*X*) of the membrane on the electrolyte concentrations. Wang et al. [32] proposed the following empirical equation (3) explaining this effect in the case of one electrolyte 1-1:

(3) $$\begin{array}{c} \Phi X=\frac{Ac^{0.5}}{1+Bc^{0.5}}. \end{array}$$

*A* and *B* are empiric constants that depend on the electrolyte and the nature of the membrane. This equation suggests that the density charge of the membrane reached a maximum at elevated salt concentration.

Using NF-HL membrane, the retention rate of Cr(VI) decreased from 91.4 to 86% in the presence of KCl and KNO_3_ and down to 74.9% by adding K_2_SO_4_, while it decreased from 98.6 to 90.2, 93.3, and 85.4% in the presence of KCl, KNO_3_, and K_2_SO_4_, respectively, in the case of RO-SG.

In presence of bivalent ions (SO_4_^2−^), the reduction in the retention rate of Cr(VI) was more pronounced than in presence of monovalent ions (Cl^−^, NO_3_^−^). This may be explained by the exclusion phenomenon attributed to the different valence of the coion.

**Figure 5:**
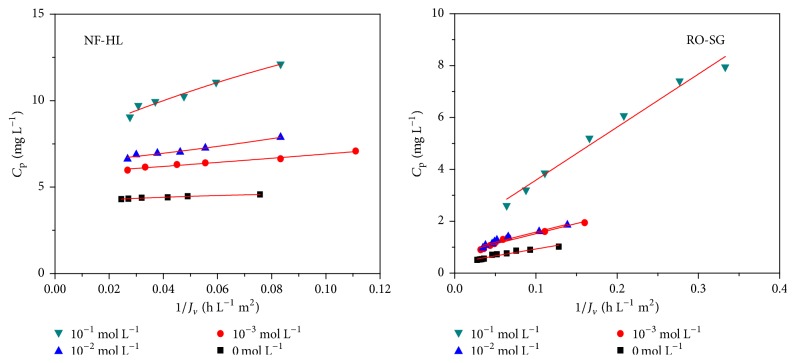
Variation of the permeate concentration in function of 1/*J*_*v*_ of Cr(VI) at different KCl concentrations (*C*_Cr(VI)_ = 50 mg L^−1^, *Y* = 50%, pH = 8, and *θ* = 25°C).

**Figure 6:**
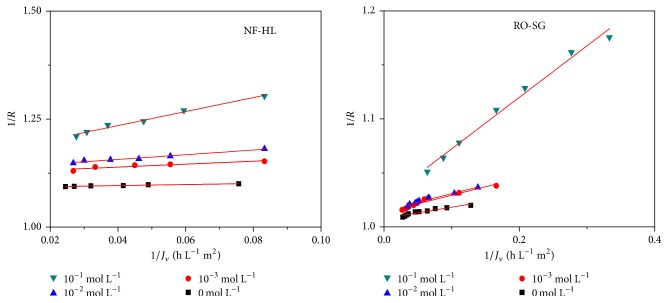
Variation of 1/R in function of 1/*J*_*v*_ for different KCl concentrations (*C*_Cr(VI)_ = 50 mg L^−1^, *Y* = 50%, pH = 8, and *θ* = 25°C).

In order to better understand the transport phenomenon, the experimental data of retention and flux for all investigated salts were fitted using the Spiegler-Kedem model to determine the salt permeability (*P*_*s*_) and reflection coefficient (*σ*) parameters as well as the diffusive and convective contributions (Figures 5 and 6).

As illustrated in Figures 5 and 6, the analysis of Cr(VI) concentration (*C*_*p*_) in the permeate in presence of KCl as a function of the reverse permeate flux (1/*J*_*v*_) revealed a linear relation in conformity with the Spiegler-Kedem model. The two membranes imply two different mechanisms of transfer of aqueous solution, both acting separately, but in an additive way on the transfer.

As illustrated in Table 2, *P*_*s*_ values seemed to be highly dependent on the nature of anion of the electrolyte solute as well as on its concentration in solution.

The presence of strongly solvated anions (SO_4_^2−^) in chromium solution leads to higher values of *P*_*s*_ in comparison with the less solvated anions (Cl^−^ and NO_3_^−^) (Table 3). *P*_*s*_ increased with salt concentration due to the high amount of hexavalent chromium passing through the membrane, while *σ* decreased due to the reduction of salt retention [33]. The reflection coefficient *σ* was higher in the presence of monovalent anions than bivalent anions. This may be due to the competition between chromium retention and the presence of anions. RO-SG membrane gives the highest values of reflection coefficient which were close to one, suggesting that the membrane gives nearly a complete rejection of hexavalent chromium and lower values of solute permeabilities compared to NF-HL membrane.

It was also noted that the diffusion part of flux was, in general, the highest in presence of SO_4_^2−^ which had the lowest diffusion coefficient as it increased with solute concentration. In addition, the convective contribution increased with solute concentration and it was higher with NF-HL than with RO-SG membrane.

Even if the experimental rejection evolution with permeation flux was well-fitted by the model, in the case of bivalent salt K_2_SO_4_, the fit was not good; an inaccurate result was obtained (*σ* > 1).

### 3.2. Removal of Cr(VI) from Car Shock Absorber Factory Effluent

In this part, the efficiency of NF-HL and RO-SG membranes in reducing the amount of Cr(VI) ions from car shock absorber factory effluent was studied. Spiegler-Kedem model was applied to fit the experimental data and evaluate the parameters *σ* and *P*_*s*_. The optimal membrane was selected on several criteria such as water permeability, removal of total salinity, and hexavalent chromium selectivity.

Industrial effluent after neutralization with HCl was collected and analyzed. The results of feed water analysis are presented in Table 4.

#### 3.2.1. Hydraulic Permeability of Wastewater

**Figure 7:**
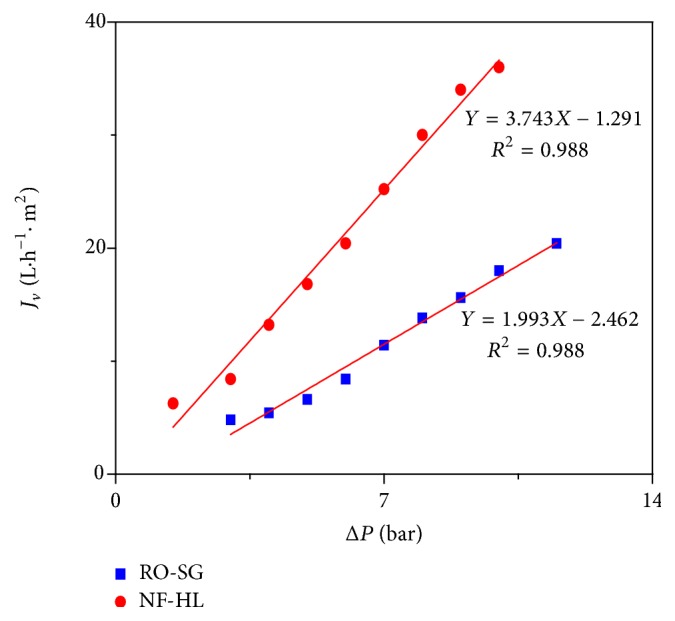
Effect of transmembrane pressure on the permeate flux of wastewater.

The experimental data for the permeate flux, with wastewater, as a function of the transmembrane pressure are given in Figure 7 for the two tested membranes.

The permeate fluxes obtained for the NF-HL membrane were higher than those of the RO-SG membrane. The wastewater permeabilities (*L*_*p*_′_NF-HL_ = 3.743 L h^−1^ m^−2^ bar^−1^, *L*_*p*_′_RO-SG_ = 1.993 L h^-1 ^m^−2^ bar^−1^) were lower than those of pure water (*L*_*p*_). The presence of the electrolyte in solution made the membrane surface more compact due to the contraction of pores, resulting in a decrease in the permeability through membranes [34].

The critical pressure *P*_*c*_ was defined as

(4) $$\begin{array}{c} P_{c}=\sigma \cdot \Delta \pi . \end{array}$$

The critical pressure of RO-SG membrane (Pc = 2.462 bar) was nearly two times higher than that of NF-HL membrane (Pc = 1.291 bar) which was due to the higher rejection and the obviously higher osmotic pressure difference across the membrane. The NF-HL membrane having more open pores was less dependent on osmotic pressure compared with RO-SG membrane because of lower retentions.

#### 3.2.2. Removal of Total Salinity

**Figure 8:**
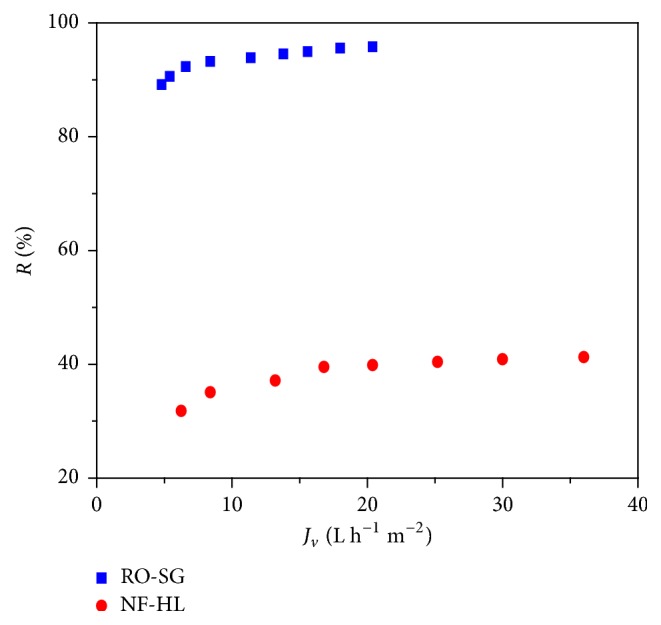
Total salinity rejection during wastewater treatment by NF-HL and RO-SG membranes, pH = 8, *Y* = 50%, and *θ* = 25°C.

The highest retentions are obtained for RO-SG membrane (>90%), while it was between 31.8 and 41.2% for NF-HL (Figure 8). There was an important effect of the screen phenomenon of the membrane charge at high concentration level, which was the case for the treated effluent.

The permeate salinity confirmed the satisfactory performances of RO-SG (249–97 *μ*S cm^−1^) for desalinating wastewater compared with NF-HL (1565–1348 *μ*S cm^−1^).

#### 3.2.3. Removal of Hexavalent Chromium

Experiments were conducted to determine the effectiveness of the two membranes for hexavalent chromium removal from wastewater and to possibly reuse the water.

**Figure 9:**
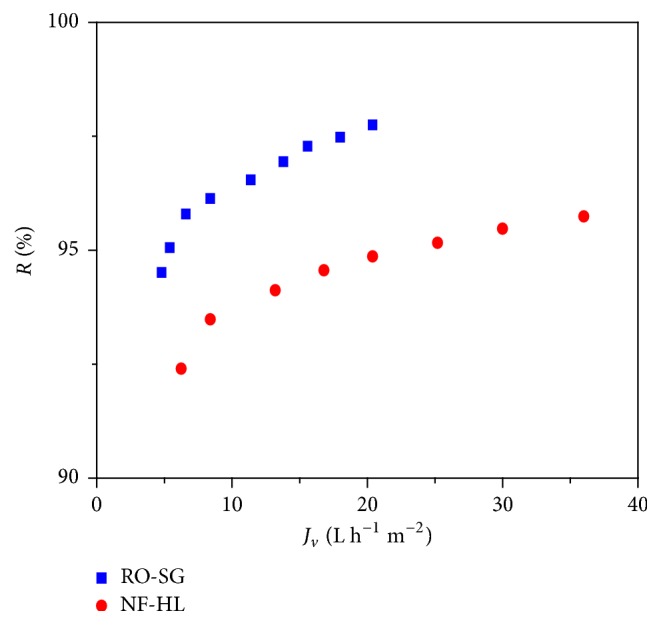
Retention rate of Cr(VI) versus transmembrane pressure for NF-HL and RO-SG membranes, *Y* = 50%, pH = 8, and *θ* = 25°C.

The results showed that the two membranes were suitable for the treatment of the Cr(VI) present in the car shock absorber factory effluent (Figure 9).

The highest retentions are obtained for RO-SG membrane (94.5–97.7%), while it was between 92.4 and 95.7% for NF-HL. The permeate Cr(VI) concentration reached 0.47 mg L^−1^ for NF-HL and 0.24 mg L^−1^ for RO-SG. These values lied in the limits recommended by National Sanitation Utility of Tunisia (ONAS).

#### 3.2.4. Modeling the Ion Rejection of Wastewater

Spiegler-Kedem model was applied to fit the rejection of total salinity and chromium ions. The fitting parameters (*σ* and *P*_*s*_) for the two membranes are given in Table 5.

*σ* and *P*_*s*_ values depended on the nature of the membrane. The RO-SG presented higher *σ* values and lower salt permeabilities compared to the NF-HL membrane. The reflection coefficient showed that the retention rate of Cr(VI) was higher than the total salinity. The model fitted well with the experimental data of the retention of both total salinity and hexavalent chromium ions for both membranes.

## 4. Conclusion

Hexavalent chromium removal was investigated using two commercial membranes (NF-HL and RO-SG). The removal efficiency for hexavalent chromium was influenced by ion concentration, pH, conversion rate, transmembrane pressure, and the presence of different coexisting ions. The Cr(VI) retention of the RO-SG membrane was higher than that of NF-HL membrane under various operating conditions and reached the standard limits. It was observed that increasing ion concentration of the feed solution retention depended on pH. The retention of chromium decreased with increasing concentration of the electrolyte support which might be explained as a result of decreasing effective charge density of the membrane surface. The reduction in membrane surface charge decreased the charge repulsion between the ions and the membrane. The experimental results showed that the use of these membranes was very promising for water treatment containing Cr(VI) with a good efficiency in desalting industry effluent. Membrane parameters (reflection coefficient and solute permeability) were shown to depend on the nature of membrane and the anions present in feed water.

## Figures and Tables

**Table 1:** Specifications of NF-HL-2514 and RO-SG-2514 membranes.

Membrane reference	NF-HL-2514	RO-SG-2514
Membrane type	Polyamide thin film composite	Polyamide thin film composite
Maximum operating temperature	50°C	50°C
Maximum operating pressure	40 bar	41 bar
Continuous operating pH range	4–11	2–11
Membrane area	0.6 m^2^	0.6 m^2^
Pure water permeability	9 L h^−1^ m^−2^ bar^−1^	3.85 L h^−1^ m^−2^ bar^−1^
MWCO	314 Da	172 Da

**Table 2:** Transport parameters (*σ* and *P*_*s*_), diffusive flux *J*_diff_ and *C*_conv_ of hexavalent chromium in presence of different salts (*C*_Cr(VI)_ = 50 mg L^−1^, *Y* = 50%, pH = 8, and *θ* = 25°C).

		*C* (mol L^−1^)	*J*_diff_ (L h^−1^ m^−2^)	SD (%)	*C*_conv_ (mg L^−1^)	SD (%)	*σ*	SD (%)	*P*_*s*_	SD (%)
KCl	NF-HL	0	5.09	0.17	4.195	0.095	0.916	0.04	0.7102	1.06
		0.001	24	0.30	4.889	0.094	0.903	0.017	1.4879	3.68
		0.01	28.17	0.06	5.088	0.01	0.901	6 10^−5^	1.6384	2.65
		0.1	27.14	0.21	6.884	0.1	0.865	5 10^−6^	1.7517	4.75

KCl	RO-SG	0	5.33	0.07	0.398	0.092	0.993	0.0095	0.168	0.06
		0.001	7.82	0.15	0.716	0.035	0.987	0.049	0.269	0.04
		0.01	7.80	0.01	0.801	0.01	0.985	0.1	0.267	1.09
		0.1	20.40	0.04	1.543	10^−4^	0.976	0.152	0.545	0.09

KNO_3_	NF-HL	0	5.09	0.09	4.195	0.004	0.916	0.38	0.7102	0.003
		0.001	11.97	0.02	5.715	0.0014	0.886	0.01	1.0366	0.09
		0.01	24.91	0.18	5.963	0.098	0.885	0.03	1.5274	1.63
		0.1	47.17	0.08	8.081	0.02	0.848	0.1	2.5262	0.08

KNO_3_	RO-SG	0	5.33	0.07	0.398	0.0003	0.993	0.03	0.168	0.007
		0.001	6.43	0.09	0.402	0.0007	0.992	0.04	0.184	0.0034
		0.01	8.43	0.04	0.509	0.0004	0.990	0.08	0.249	0.002
		0.1	12.52	0.03	1.739	0.0013	0.968	0.01	0.388	0.09

K_2_SO_4_	NF-HL	0	5.09	0.08	4.195	0.015	0.916	4 10^−3^	0.7102	0.008
		0.001	12.80	0.10	6.971	0.1	0.869	0.25	1.2333	0.001
		0.01	32.157	0.01	6.801	0.001	0.871	0.145	1.8242	0.001
		0.1	116.83	0.18	7.780	0.005	nd	nd	nd	nd

K_2_SO_4_	RO-SG	0	5.33	0.11	0.398	0.00023	0.993	0.049	0.168	1.78
		0.001	9.82	0.1	0.700	10^−4^	0.986	0.01	0.314	1.02
		0.01	8.24	0.2	0.884	0.0005	0.982	0.07	0.293	0.008
		0.1	21.77	0.2	2.746	1.14 10^−5^	0.957	0.23	0.652	0.09

**Table 3:** Hydration energies, hydrated radii, and diffusion coefficients of some ions [30, 31].

Ion	Na^+^	K^+^	Cl^−^	NO_3_^−^	SO_4_^2−^
Hydration energies (kJ mol^−1^)	454	363	325	310	1047
Hydrated radii (nm)	0.358	0.331	0.332	0.335	0.379
*D* (10^9^ m^2^·s^−1^)	1.333	1.957	2.032	1.902	1.065

**Table 4:** Water quality of car shock absorber factory before and after neutralization and comparison with Tunisian standards liquid discharges into the network “National Office for Sanitation” (ONAS) (NT 106-02).

	Wastewater before neutralization	Wastewater after neutralization	Tunisian standards (NT 106-02)
*T* (°C)	24	24	<35
pH	13.45	8	—
Conductivité (*µ*S cm^−1^)	3023	3027	—
TDS (mg L^−1^)	2293	2296	—
Turbidité (NTU)	2.08	0	—
Cr(VI) (mg L^−1^)	11.06	11.06	0.5

**Table 5:** Reflection coefficients (*σ*) and solute permeabilities (*P*_*s*_) for total salinity and Cr(VI) ions of treated wastewater by NF-HL and RO-SG membranes.

Membrane	*C* (mg L^−1^)	*σ*	SD (%)	*P*_*s*_	SD (%)
NF-HL	Cr(VI)	0.962	0.01	0,5356	0.09
	Salinity	0.443	1.04	6,2616	2.78
RO-SG	Cr(VI)	0.985	0.03	0,2744	0.01
	Salinity	0.977	0.07	0,5497	1.23
